# VariGAN: Enhancing Image Style Transfer via UNet Generator, Depthwise Discriminator, and LPIPS Loss in Adversarial Learning Framework

**DOI:** 10.3390/s25092671

**Published:** 2025-04-23

**Authors:** Dawei Guan, Xinping Lin, Haoyi Zhang, Hang Zhou

**Affiliations:** School of Electronic and Information Engineering, Beijing Jiaotong University, Beijing 100044, China; 22721006@bjtu.edu.cn (D.G.); 22721018@bjtu.edu.cn (X.L.); 22721074@bjtu.edu.cn (H.Z.)

**Keywords:** style transfer, GAN, LPIPS, UNet, ResNet, image segmentation

## Abstract

Image style transfer is a challenging task that has gained significant attention in recent years due to its growing complexity. Training is typically performed using paradigms offered by GAN-based image style transfer networks. Cycle-based training methods provide an approach for handling unpaired data. Nevertheless, achieving high transfer quality remains a challenge with these methods due to the simplicity of the employed network. The purpose of this research is to present *VariGAN*, a novel approach that incorporates three additional strategies to optimize GAN-based image style transfer: (1) Improving the quality of transferred images by utilizing an effective UNet generator network in conjunction with a context-related feature extraction module. (2) Optimizing the training process while reducing dependency on the generator through the use of a depthwise discriminator. (3) Introducing LPIPS loss to further refine the loss function and enhance the overall generation quality of the framework. Through a series of experiments, we demonstrate that the *VariGAN* backbone exhibits superior performance across diverse content and style domains. *VariGAN* improved class IoU by 236% and participant identification by 195% compared to *CycleGAN*.

## 1. Introduction

Generative artificial intelligence demonstrates significant application potential across various fields [1,2,3]. In the field of education, automated assessment tools are employed to enhance teaching effectiveness. In the arts and creative industries, generative AI is utilized to generate music, paintings, and writing, inspiring novel methods of creation and artistic expression. In recent years, the task of image style transfer has garnered significant attention [4,5,6,7,8,9]. Among these, generative models based on generative adversarial networks (GANs) represent one of the most prominent areas of contemporary research. Content images and style images are fed into a trained generative network, which produces an image that undergoes style transfer while preserving the relevant content. Most methods focus on the training of paired images [5,7,10,11,12]. Since training content must be a one-to-one correspondence, the generation ability of this network is poor. To effectively train the model, the majority of traditional style transfer techniques require paired images, which restricts their applicability to new datasets and content. The model’s versatility is limited by its reliance on paired data, and because content and style do not always correlate one to one, it frequently performs poorly when dealing with heterogeneous, unstructured data.

In order to solve the problem of training unpaired datasets, *CycleGAN* [8] proposes a method of loop training, which uses the generator to transform from the source domain to the target domain for discrimination and then re-transform to the source domain. It can achieve the content style transfer of unpaired images. *StyleGAN* [13,14,15] uses a multilayer perception for connection and an encoder–decoder structure [16] for feature extraction and transmission, which solves the problem of artifacts in the generation effect of the cycle training mode to some extent. *DualGAN* [17] and *DiscoGAN* [18] have contributed to the advancement of unpaired image style transfer. *DualGAN* ensures that the training of the GAN model is more stable by introducing the strategy of dual learning, effectively reducing the mode collapse problem. *DiscoGAN* can selectively perform style transfer. However, they cannot integrate style characteristics and content characteristics well. The diffusion model [19] has emerged as one of the most competitive generative models in recent years. While diffusion models for style transfer effectively preserve both style and content characteristics, owing to their high fidelity in details they often lead to excessive editing of the semantics outside the content body [20,21]. In this project, we aim to address the generation quality issues using GAN-based approaches.

However, the framework mentioned above still has the problems of poor generative ability and low robustness. Owing to the constrained architecture of the *CycleGAN* generator network, its capacity to capture image edges and high-frequency details is insufficient. The discriminator exhibits slow convergence speed, and its generalization ability on unseen samples is limited. In order to solve the above problems, we build an image style transfer model which has high-quality generation capability and can be generalized. We propose our model *VariGAN*, which focuses on optimizing the generators and discriminators in the GAN model [22]. To enhance the generative capabilities of the model, we use the UNet network, which combines self-focused modules and global ResNet blocks as the generator. The generation process is optimized by increasing the network depth and modifying the feature extraction approach from simple convolution. By employing deep convolutional networks as discriminators, the dependence between the generator and discriminator can be weakened, the convergence speed of the discriminator can be improved, and the training process can be further optimized. In the selection of the loss function, a loss function that closely aligns with human perception is selected as the control quantity for the transfer between sample domains, which can further assist the generator in producing high-quality outputs. Compared with previous methods, our method emphasizes optimizing the extraction and transmission of image content and stylistic features. The network can extract more necessary information and enhance the quality of generated outputs. Finally, the problems existing in the work are analyzed, and future research directions are outlined.

## 2. The Contributions of Our Work

- We use a UNet generator with a self-attention mechanism to enhance detail processing and a global ResNet block to fuse attention features (*Q*, *K*, *V*).
- We introduce the depthwise discriminator to refine and stabilize the training process.
- We incorporate LPIPS loss to improve the perceptual similarity in the loss function.
- Detailed qualitative and quantitative evaluations validate the efficacy and robustness of our *VariGAN*.

## 3. Related Work

### 3.1. Style Transfer of Images

Image style transfer has transitioned from static task definitions to dynamic, concept-driven methodologies. The definition of content loss and style loss initiated the process of image style transfer [4]. In 2017, Huang [5] showed that network-extracted features encapsulate style, significantly improving the learning efficiency and speed of the network. However, the use of simple convolution networks constrained the quality of generated results. GANs [6,23] introduced the ability for generators to learn mappings from noisy spaces to target image domains, while *CycleGAN* [8] enhanced the practicality of neural networks in style transfer by enabling training on non-matching image pairs. Despite these advancements, GANs still face challenges like mode collapse and instability during training [24], which limit their generative ability.

Recent advancements have sought to overcome these limitations. For example, Puff-Net [25] introduces a lightweight and efficient architecture for style transformation, balancing quality, speed, and structural integrity. Adaptive Instance Normalization (AdaIN) [5], a pivotal technique in style transfer, aligns the mean and variance of content features with those of style features, enabling fast and flexible stylization. Recent extensions of AdaIN have aimed to enhance style consistency and adaptability to diverse input styles, further improving its utility in real-time applications. In addition, diffusion models [26] have emerged as a powerful tool for style transfer and portrait generation, leveraging disentangled representations and semantic guidance to produce high-quality, controllable outputs. In the area of 3D image editing, *InstantStyleGaussian* [27] introduced a 3D Gaussian splash representation, enabling fast and effective style transfer in 3D environments.

Our work addresses the challenges of low generation quality and poor robustness in GAN-based style transfer. While encoder–decoder structures have been widely adopted to address issues like uncontrollable discretization in the target domain [14], their limited convolutional depth often leads to incomplete style transfer and noisy outputs. In this paper, we present our approach to these challenges, combining insights from recent advancements and novel techniques, as described in Section 4.

### 3.2. Semantic Segmentation Model Based on Deep Learning

Using a fully convolutional network (FCN) [28], the semantics of the image only depends on the convolution layer style, and segmentation images of any size can be produced. Through deep convolutional networks, end-to-end semantic segmentation capability can be realized. However, there are still challenges in extending these methods to 3D images, as high-dimensional data require more robust architectures. Probability graph models [29] have become a promising solution, by capturing the global context and improving the segmentation accuracy to resolve the depth of the limitations of CNNs. These models integrate spatial dependencies into the network for better semantic understanding, especially in complex scenarios. Encoder–decoder model such as UNet [30] have had a wide range of success in medical image segmentation, retaining the mode of information and at the same time providing a balance of convolution and FCN depth. The use of specific areas and mask [31,32,33] has been studied. Convolutional neural networks can focus on detailed semantic information, enhance global image understanding, and achieve improved segmentation performance. In order to solve some limitations of traditional UNet in image segmentation, scholars have used many ways to optimize the traditional UNet network. MultiResUNet [34] improves the performance of UNet in multi-scale image segmentation by introducing ResNet logic. Brau-net++ [35] improves the performance of complex image segmentation by introducing dual-path attention to learn local semantic information. Sharp UNet [36] introduces the logic of deep convolution, which can effectively merge semantically similar features and improve network performance while maintaining computational load. This idea of using deep separation convolution is very similar to our idea of optimizing the discriminators.

Building on the success of these methods, our work incorporates an FCN to evaluate 2D images and employs three FCN-based metrics to quantitatively evaluate the performance of our method. These metrics enable robust analysis of *VariGAN*’s effectiveness in addressing the challenges of style consistency and semantic segmentation.

### 3.3. Encoder–Decoder Architecture

Autoencoders inspired the development of encoder–decoder networks, which are now essential for tasks like image generation and translation. To align with the target domain, this architecture compresses input data into a latent space before reconstructing them [37]. The inability of early encoder–decoder models to preserve detail was addressed by UNet [38], which introduced skip-connections to retain high-resolution features commonly used in segmentation tasks. Johnson [39] demonstrated that an encoder–decoder with perceptual loss could effectively transfer styles while preserving content. By learning pixel-level mappings across domains, Pix2Pix [7], a GAN with an encoder–decoder generator, further advanced image translation capabilities. However, these models still face challenges like mode collapse and poor style representation, particularly when dealing with complex textures.

To address these issues, Zhang [40] introduced attention techniques that enable the encoder–decoder to focus on key features, improving detail and reducing noise. Attention mechanisms combined with encoder–decoder architectures were introduced to enhance texture management. However, challenges such as texture discretization and noise generation remain [41]. Inspired by UNet, we employ a UNet network combined with a self-attention module to optimize the generator network. We outline our strategy to overcome these constraints in Section 4.

### 3.4. CycleGAN

*CycleGAN* (Cyclic Generative Adversarial Network) [8] is an unsupervised image style transfer model that employs two generators and two discriminators to facilitate image conversion between source and target domains.

The *CycleGAN* architecture consists of two generators: *G* and *F*. Generator *G* transforms images from the source domain (*X*) to the target domain (*Y*), while generator *F* maps images from the target domain (*Y*) back to the source domain (*X*). Additionally, *CycleGAN* includes two discriminators: $D_{Y}$, which evaluates whether an image generated in the target domain is real; and $D_{X}$, which assesses the authenticity of images generated in the source domain.

**Figure 1:**
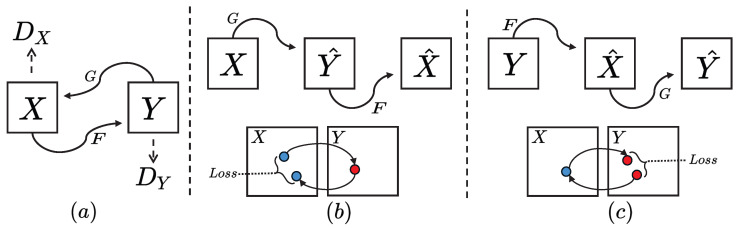
(**a**) Two mapping functions, G:X⟶Y and F:Y⟶X, along with their corresponding adversarial discriminators, $D_{Y}$ and $D_{X}$, are incorporated into our model. The discriminator $D_{Y}$ encourages *G* to map *X* to outputs indistinguishable from domain *Y*, while $D_{X}$ and *F* perform a similar function for domain *X*. (**b**) The logic of sample domain migration is $X⟶\hat{Y}⟶\hat{X}$. (**c**) The logic of sample domain migration is $Y⟶\hat{X}⟶\hat{Y}$. To further regularize the mapping, we not only use two cycle consistency losses but also incorporate LPIPS loss into the loss function. This ensures that transforming from one domain to another and then back again returns the input to its original domain. The blue and red dots represent the samples in the domain.

For the forward transformation process, a source-domain image *x* is first converted into a target-domain image $y^{\prime }$ by generator *G*. This generated image $y^{\prime }$ is then reconstructed back into a source-domain image $x^{\prime }$ using generator *F*. Similarly, for the reverse transformation, the roles of *G* and *F* are interchanged. A schematic representation of this training process is provided in Figure 1.

However, as the generation network primarily relies on convolutional layers, crucial style features may be lost during the transformation process, leading to suboptimal generation results. To address this limitation, our method introduces enhancements to preserve style features during image conversion, as detailed in the following sections.

For the loss function in training, *CycleGAN* uses two types of loss working together. Cyclic consistency loss: By calculating the difference between the source-domain image *x* and the image obtained after bidirectional conversion $x^{\prime }$, the cyclic consistency loss [42] is obtained:

(1) $$L_{\mathrm{cycle}\;}\left(G,F\right)={∥F\left(G\left(x\right)\right)-x∥}_{1}+{∥G\left(F\left(y\right)\right)-y∥}_{1}$$

For the discriminator $D_{Y}$, the goal is to maximize the probability of discriminating the real object domain image and generating the image:

(2) $$L_{D_{Y}}=-E\left[\log D_{Y}\left(y\right)\right]-E\left[\log\left(1-D_{Y}\left(G\left(x\right)\right)\right)\right]$$

The generation quality of *CycleGAN* at the level of human-eye perception is still insufficient, and the image consistency is poor. We have improved the training loss function, which is explained in Section 4.

## 4. Methodology

Although GANs can achieve iterative learning to complete the task of style transfer, the quality of image generation is often poor due to the problem of GANs’ innate pattern collapse [24]. The generated image may also have too many or too few style features, edge artifacts, etc. In order to improve the generating effect of the network, we have made the following improvements in our *VariGAN* network: (1) The UNet network combined with a self-attention module is used as a generator to improve the feature transmission process and improve the quality of the generated image. (2) The discriminator uses a depthwise structure, changing the disadvantage of the discriminator’s dependency on the generator. (3) Based on the inspiration of human-eye perception consistency [43], we use LPIPS loss as a penalty on the basis of the original *CycleGAN* to improve the optimization degree of the generative network.

### 4.1. UNet-Like Generator with Self-Attention Block

In the generator, inspired by Chen [44], a UNet framework is adopted to replace the original encoder–decoder network [45]. Enhancing the network depth and feature extraction mechanisms improves the network’s ability to disentangle long-distance image features and complex spatial structures, thereby improving the quality of image generation more effectively. To this end, the generator incorporates a self-attention mechanism to enhance feature extraction while incorporating UNet’s skip-connection mechanism to increase the network depth.

**Figure 2:**
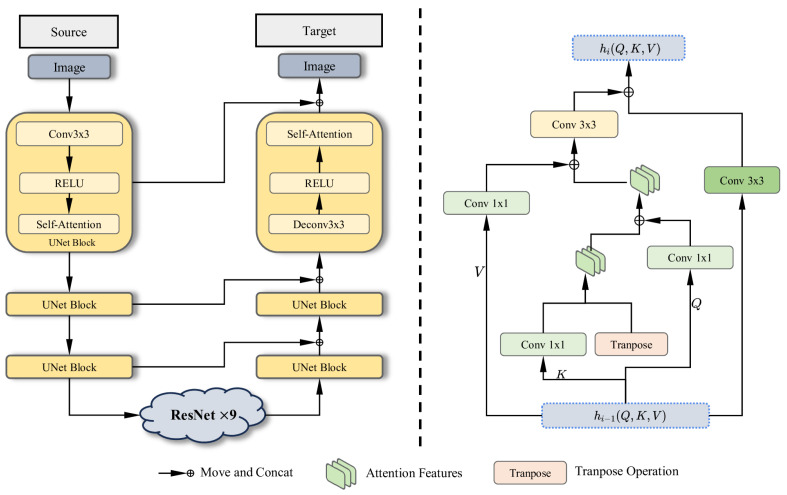
Overview of the generator. On the left is the basic framework of the generator, which is a UNet-like framework that combines a self-attention module and a globally connected residual network. On the left is the residual network used in the bottleneck. It optimizes feature transfer by handling fusion of *Q*, *K*, and *V*, respectively. In this symmetric structure, there are three layers for both downsampling and upsampling. In each layer, the framework uses a convolution layer, a self-attention layer, and a ReLU activation function. Specific legends are included below for concat operations, attention features (*Q*, *K*, *V*), and transpose operations.

The original encoder–decoder network is a three-layer convolutional sampling network, which performs three downsampling operations followed by deconvolution to reconstruct an image of the same size as the original. For our generator network, a 3-layer UNet [30] is adopted and the input shape is (256,256,3), with each layer comprising a convolutional layer, a self-attention layer [46], and an activation function, as shown in Figure 2. In the bottleneck part of the structure, a 9-layer global ResNet residual connectivity network [47] is employed.

Meanwhile, the idea of skip-connections [48] is applied to fuse features of the same scale from the encoder into the decoder, thereby enhancing the quality of the generated images. The specific transfer formula is shown below.

(3) $$y_{i}=SelfAttention(RELU\left(Conv3\left(y_{i-1}\right))\right)$$

where $y_{i}$ represents the output of the current layer, and $y_{i-1}$ represents the output of the previous layer. i=2,3 and $y_{0}$ represents the input content images. Conv3() is a 3×3 convolution block, SelfAttention() is the self-attention module [49,50], and RELU() is the activation function [51]. The specific configuration is inplace=True.

The self-attention mechanism computes attention scores based on the representations of the query (*Q*), key (*K*), and value (*V*), derived from the input sequence via linear transformations. Specifically, the query representation $Q=XW_{Q}$ is formulated, where *X* is the input sequence and $W_{Q}$ is the learned weight matrix for queries. Similarly, the key representation is given by $K=XW_{K}$, where $W_{K}$ is the learned weight matrix for keys, and the value representation is $V=XW_{V}$, where $W_{V}$ is the learned weight matrix for values.

To reduce computational complexity, a downsampling step is introduced prior to computing the attention. Specifically, the input feature map *X* is downsampled using nearest-neighbor interpolation by a factor of the reduction ratio (r=16). This results in a smaller feature map $X_{\mathrm{reduced}}$ with dimensions $H^{\prime }\times W^{\prime }$, where $H^{\prime }=H/r$ and $W^{\prime }=W/r$, which effectively reduces the spatial resolution of the input. This downsampling ensures that the attention mechanism operates on a smaller spatial dimension, significantly reducing computational cost without losing critical spatial information. After attention scores are computed, the output is restored to the original resolution via interpolation.

The specific formula for the self-attention mechanism remains as

(4) $$\mathrm{SelfAttention}\left(Q,K,V\right)=\mathrm{softmax}\left(\frac{{\mathrm{QK}}^{T}}{\sqrt{d_{k}}}\right)V$$

where $d_{k}$ is the dimensionality of the key vectors, ensuring proper scaling of the dot product. The attention mechanism computes a weighted sum of the value vectors using the calculated attention scores. However, by applying downsampling to the input *X*, both the time and space complexity are reduced, making the attention mechanism more efficient.

A residual connection is incorporated after the attention operation. Instead of directly outputting the result from the attention mechanism, the original input *X* is added back to the output, scaled by a learnable parameter γ. This residual connection facilitates improved gradient flow during backpropagation while preserving the original input information, allowing for more effective learning. The output out after attention is computed as

(5) out=γ·Attention(Q,K,V)+X

where γ is a learnable scaling parameter and is initialized as 0.05, which allows the model to adaptively control the influence of the residual. This modification improves the model’s capacity to learn intricate representations while retaining essential features from the input.

For the decoding part, we imply the skip-connection mechanism; the specific formula is shown below.

(6) $${y_{i}}^{\prime }=SelfAttention(RELU\left(Deconv\left(Concat\left({y_{i-1}}^{\prime },y_{i-1}\right)\right))\right)$$

where ${y_{i}}^{\prime }$ represents the output of the current layer, and ${y_{i-1}}^{\prime }$ represents the output of the previous layer. *i* can take the value 2 or 3.

As for why we use UNet with a 3-layer encoder–decoder structure as our generator network, this is a choice we made based on experience. In fact, we also analyze the number of layers in the generator network in Section 5.

It is possible for us to keep a significant amount of the content information of the image by utilizing the generator network described above. When it comes to the process of style transfer, the issues of pattern collapse and the general generation effect of the GAN model are successfully regulated, and the quality of the generation is effectively improved.

### 4.2. Globally Connected ResNet Block

The globally connected ResNet is located in the bottleneck part of the UNet, as shown in Figure 2. Inspired by Zhang [52], we employ a global residual-linked network to enhance the transmission of deep image features. The input to this module consists of the self-attention features *Q*, *K*, and *V* extracted from the downsampling part of the UNet.

To effectively integrate self-attention into the ResNet framework, we first process *Q*, *K*, and *V* separately to retain their individual contributions. The K matrix undergoes a 1×1 convolution, and then is transposed and concatenated with itself to form $K^{\prime }$, which encodes the refined key information. The updated $K^{\prime }$ is then concatenated with *Q* and *V*, each of which has been independently processed through a 1×1 convolution block. This ensures that self-attention features are fully incorporated into the ResNet structure without significant information loss. Finally, the processed features, along with the output from the previous ResNet block, are concatenated and passed through a 3×3 convolution before being fed into the next ResNet layer. This integration allows the self-attention module to efficiently guide feature refinement while leveraging the global feature learning capability of the ResNet structure. The corresponding formula is provided below.

(7) $$\begin{array}{cc} h_{i}= & \mathrm{Concat}\left(\mathrm{Conv}3\left(h_{i-1}\right),\right.\\ & Conv3(\mathrm{Concat}(Conv1(V)),\\ & \left.\left.\mathrm{Concat}\left(Conv1\left(Q\right),\mathrm{Concat}\left(K,K^{T}\right)\right)\right)\right) \end{array}$$

where $h_{i-1}$ represents the output of the previous layer, and $h_{i}$ represents the output of the current layer, where i=1,2,3. Concat() stands for the concat operation, Conv3() stands for the 3×3 convolution block, and Conv1() stands for the 1×1 convolution block. $K^{T}$ is the transition matrix of *K*.

This global connection mode of ResNet not only effectively ensures that the output of the self-attention module is effectively transmitted, but also reduces the loss of attention features (Q,K,V) in the convolution. Thus, the generation ability of the generator network is improved.

### 4.3. Depthwise Discriminator with Depthwise and Pointwise Convolution

The proposed discriminator architecture employs depthwise separable convolution to address the limitations of conventional GAN frameworks, in which the discriminator’s capacity is often restricted by the generator’s performance. The use of depthwise separable convolution in the discriminator enhances the detection of edge details during the discrimination process. It also reduces specks or artifacts generated at the edges, thereby improving the overall quality of the generated images.

**Figure 3:**
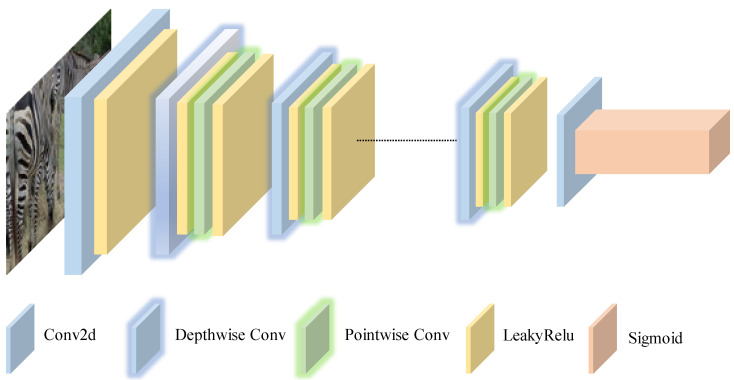
Schematic diagram of the discriminator: The discriminator architecture begins with a standard convolution layer, followed by a series of depthwise and pointwise convolution layers, and concludes with a global convolution layer to evaluate the image generated by the generator. The first six convolutional layers extract image features at different resolutions, while the global convolution layer performs the final evaluation. This structure reduces computational complexity and improves both recognition efficiency and accuracy.

As illustrated in Figure 3, the network adopts a hierarchical feature extraction strategy through six successive depthwise separable convolution blocks, which facilitates the simultaneous optimization of the discriminator and generator during adversarial training. This structural innovation substantially improves the model’s capacity to capture high-frequency details in 256×256-resolution images, all while ensuring computational efficiency.

The network starts with a standard convolution layer with a 4×4 convolution kernel for initial downsampling, with an input shape of (256,256,3) and an output shape of (16,16,1). Subsequent stages employ four depthwise separable convolutions with progressive channel expansion, facilitating feature extraction at various scales. Each block performs spatial filtering and channel feature recombination through two sequential operations: (1) depthwise convolution with instance normalization and LeakyReLU; (2) pointwise convolution with similar normalization and activation functions. The final layer uses a 16×16 convolution kernel to achieve global feature aggregation, which is analogous to a fully connected layer but retains spatial correlation.

The formulation of the standard convolution layer is given by

(8) $$C_{1}\left(t\right)=\mathrm{IN}\left(W_{1}*t+b_{1}\right),\;L\left(x\right)=\mathrm{LeakyReLU}\left(x,0.2\right)$$

where $W_{1}\in R^{4\times 4\times 3\times 64}$ is the convolution kernel and IN represents instance normalization. ∗ represents the normal convolution operations. The negative slope of the LeakyReLU() is set to be 0.2.

For the depth-separable convolution block (layer *i*, i=2−6), the formula is as follows:

(9) $$\begin{array}{cc} D_{i}^{\left(dw\right)}\left(x\right) & =L\left(\mathrm{IN}\left(W_{i}^{dw}⊛x\right)\right),\;W_{i}^{dw}\in R^{3\times 3\times C_{in}\times 1}\\ D_{i}^{\left(pw\right)}\left(x\right) & =L\left(\mathrm{IN}\left(W_{i}^{pw}*x\right)\right),\;W_{i}^{pw}\in R^{1\times 1\times C_{in}\times C_{out}} \end{array}$$

⊛ represents a channel-independent convolution operation, and $C_{in}$ and $C_{out}$ represent the number of input and output channels.

For the global discriminant layer,

(10) $$F_{out}=\sigma \left(\sum_{k=1}^{16}\sum_{l=1}^{16}W_{f}\left[k,l\right]\cdot C_{6}^{\left(16-k,16-l\right)}\left(t\right)\right)$$

where $W_{f}\in R^{16\times 16\times 512\times 1}$ is the full receptive field convolution kernel, and $C_{6}^{\left(m,n\right)}$ represents the value of the sixth-layer feature map at position (m,n). σ represents the sigmoid activation function, which maps the eigenvalues to the interval [0,1], representing the trustworthiness of each pixel.

This optimized discriminator can effectively reduce the number of parameters in the network, which is verified in Section 5.

### 4.4. Loss Function

The GAN loss and perceptual loss utilized by *CycleGAN* are retained. In addition, the cycle consistency loss is replaced with the LPIPS loss. The LPIPS loss emphasizes human perception of image quality. Penalties aligned with human perception are introduced during the conversion process between the two sample domains, thereby effectively constraining the GAN networks and mitigating excessive migration or degraded quality during style transfer.

Although the cycle consistency loss ensures the pixel-level consistency of the image during the transformation of the two domains, the LPIPS loss pays more attention to the perceptual similarity, which is more in line with human visual perception. By using the LPIPS loss, we aim to improve the perceptual quality of the generated images to make them more visually realistic and natural. This change also allows the model to focus more on high-level features in the generated image, rather than strictly maintaining pixel-wise consistency.

In order to extract features, the main idea is to enter the real picture *x* and the image to be tested $x_{0}$ into the network *F* [53]. Then, in different channels, determine the distance between the features of *x* and $x_{0}$. Feature stacks are normalized across various channels after being extracted in various convolution layers. The result at this point is denoted as ${\hat{y}}^{L},{\hat{y}}_{0}^{L}\left({\hat{y}}^{L},{\hat{y}}_{0}^{L}\in R^{H_{L}\times W_{L}\times C_{L}}\right)$. The channel is activated by scaling the vector $w^{L}\;\left(w^{L}\epsilon R^{C_{L}}\right)$, averaging the distance in space using the L2 norm, and summing over the channel to calculate $d_{0}$. The formula is as follows:

(11) $$d\left(x,x0\right)=\sum_{L}\frac{1}{H_{L}\;W_{L}}\sum_{h,w}{∥\mathrm{wl}\Theta \left({\hat{y}}_{\mathrm{hw}}^{L}-{\hat{y}}_{0\mathrm{hw}}^{L}\right)∥}_{2}^{2}$$

where $W_{L}$ is equivalent to the result calculated by the cosine distance formula. Finally, $d_{0}$ and the real $d_{1}$ are passed to the model containing two *RELU* FC layers with 32 channels, a single-channel FC layer, and a sigmoid layer for training, and the similarity loss function is as follows:

(12) $$\begin{array}{cc} L_{\mathrm{LPIPS}}\left(x,x_{0},x_{1},h\right)= & -h\log G\left(d\left(x,x_{0}\right),d\left(x_{1},x_{1}\right)\right)\\ & -\left(1-h\right)\log\left(1-G\left(d\left(x,x_{0}\right),d\left(x,x_{1}\right)\right)\right) \end{array}$$

In summary, the loss function used by the training framework is as follows:

(13) $$L_{\mathrm{Loss}}=\alpha L_{\mathrm{LPIPS}}\left(x,x_{0},x_{1},h\right)+\beta L_{\mathrm{GAN}}\left(G,F\right)$$

where α and β are related weights; we can adjust the style strength by adjusting different weights during training. In this work, α and β are both 0.5.

In light of this, our method brings the generated images closer to the intuitive perception of the human visual system, thereby enhancing the quality of the generated images. The following section presents specific experiments, both qualitative and quantitative in nature.

## 5. Experiments

We carry out experiments in Section 4 to confirm the efficacy of our approach. We describe the experimental measures, datasets, and training parameters. Additionally, we carry out both quantitative and qualitative experiments.

### 5.1. Implementation Details

The experiments were conducted using the *PyTorch* (Version: 2.2.2) framework on an Nvidia RTX 4090 GPU (Nvidia, Santa Clara, CA, USA). The Adam optimizer [54] was employed to facilitate optimal learning, using a learning rate of 0.003 and the Adam parameter $\beta_{1}=0.5$, $\beta_{2}=0.999$. Training was conducted for 200 epochs for each dataset. Between the 100th and 200th epochs, a learning rate scheduler LambdaLR [55] was used, which gradually reduced the learning rate to zero starting from the 100th epoch.

During training, the batch size was set to 1. In order to ensure consistency with *CycleGAN* and more effectively analyze the actual performance of our improvements, the weights α and β in the loss function were both set to 0.5. The initialization parameter γ for the residual connection of the attention layer was set to 0.05.

### 5.2. Datasets and Evaluation Metrics

We use a variety of unpaired image styles to train our network. To ensure a fair comparison, we use the same dataset as *CycleGAN*, including apples and oranges, navigation and satellite maps, etc. Table 1 provides detailed information about the style and content of the dataset, the sizes of the training and test sets, and the image dimensions in the dataset. The image preprocessing pipeline includes resizing, random cropping, horizontal flipping, conversion to tensors, and normalization. First, we magnify the image to 1.12 times its original size, then randomly crop it to 256×256 pixels, apply partial random inversion, and normalize it using the mean (0.5,0.5,0.5) and standard deviation (0.5,0.5,0.5). These transformations are commonly used for data augmentation and standardization to improve model generalization capabilities during training.

In this experiment, we employed three evaluation metrics: the FCN score, participant identification, and BRISQUE (Blind/Referenceless Image Spatial Quality Evaluator). FCNs are predominantly utilized in semantic segmentation tasks. In our work, we employ the FCN score as an evaluation metric to assess the degree of fitting between the migrated image and the real sample domain during the process of image migration. For this reason, we need to ensure that the content of the migrated image closely resembles the real sample. The FCN score is typically used to assess the performance of a fully convolutional network (FCN) [56,57,58] in image generation tasks. Evaluating model performance in these tasks typically involves measuring the similarity between the segmentation results of the generated images and the ground truth. In our experiments, the FCN score is quantified using the class IoU score.

(14) $$\;\mathrm{Class}\;\mathrm{IoU}\;=\frac{1}{N}\sum_{i=1}^{N}\frac{\left|P_{i}\cap G_{i}\right|}{\left|P_{i}\cup G_{i}\right|}$$

*N* is the number of images generated by this model per class. $P_{i}$ and $G_{i}$ are the predicted and true segmentation regions of each image.

Furthermore, we considered two other important metrics: Per-pixel accuracy and per-class accuracy. The per-pixel accuracy measures the proportion of correctly classified pixels over the total number of pixels, offering a comprehensive accuracy measure across all classes. It is defined mathematically as

(15) $$\mathrm{Per}\text{-}\mathrm{pixel}\;\mathrm{Accuracy}=\frac{\sum_{i}\mathrm{TP}i}{\sum i({\mathrm{TP}}_{i}+{\mathrm{FP}}_{i})}$$

where ${\mathrm{TP}}_{i}$ represents the true positives for class (*i*), and FPi represents the false positives for class (*i*).

The per-class accuracy, on the other hand, evaluates the average accuracy across different classes, reflecting the model’s performance on each class individually. It is given by

(16) $$\mathrm{Per}\text{-}\mathrm{class}\;\mathrm{Accuracy}=\frac{1}{M}\sum {i=1}^{M}\frac{{\mathrm{TP}}_{i}}{{\mathrm{TP}}_{i}+{\mathrm{FN}}_{i}}$$

where *M* is the total number of classes, ${\mathrm{TP}}_{i}$ is the true positives for class (*i*), and ${\mathrm{FN}}_{i}$ is the false negatives for class (*i*).

Higher scores indicate better interpretability and quality of the generated image. Typically, an FCN (fully convolutional network) is employed to generate a segmentation map [28,59,60], which is the output image generated through pixel-level classification performed by a deep learning model, where each pixel is assigned a specific category label. The output image retains the same dimensions as the original input image, but each pixel’s color or label denotes its corresponding category. The FCN semantic segmentation map provides insights into the quality and interpretability of the generated image.

Participant recognition refers to the ability of individuals to discern whether a given image has been generated by a neural network and to assess its authenticity based on human perception. Twenty-five participants from diverse groups, including individuals with professional knowledge of art and those without such expertise, were recruited [7]. Images exhibiting the styles of renowned artists were excluded to reduce the impact of prior knowledge on participants’ judgments. Participants viewed one image at a time and were asked to determine whether it was a synthetic image generated by the neural network. This process was repeated 100 times, with ratings ranging from 0% to 100%.

BRISQUE (Blind/Referenceless Image Spatial Quality Evaluator) [61] is a no-reference image quality assessment method. It evaluates image quality based on the spatial statistical characteristics of natural images by analyzing the local statistical features of images in the spatial domain. It does not need the original reference image. It uses the support vector regression model to train and predict image quality and is widely applied in quality assessment tasks such as image compression, enhancement, and denoising. The lower the BRISQUE score is, the better the image quality is.

### 5.3. Qualitative Experiment

**Figure 4:**
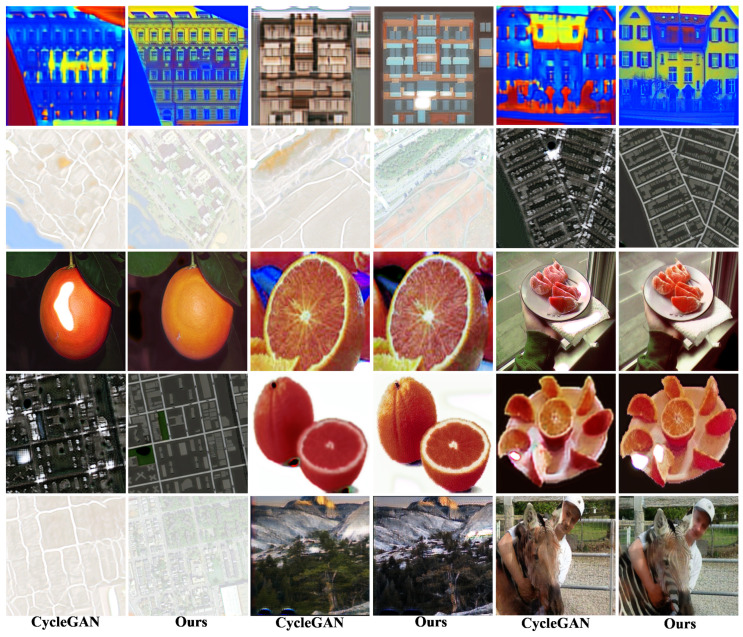
Qualitative experimental results: Comparison with *CycleGAN*. *CycleGAN* is significantly less effective than *VariGAN* in achieving style transitions. In contrast, the images produced by *CycleGAN* are not as detailed as *VariGAN*’s, nor are they as stylistically controlled.

In this section, we examine the similarities and differences between our approach and the *CycleGAN* model. Figure 4 provides a detailed comparison. For the generation of style transfer images, *CycleGAN* often fails to effectively align the style of the generated image with the content of the target domain. In contrast, our approach effectively integrates both content and style information. The results of some sets of images demonstrate that *VariGAN* generates significantly higher-quality images compared to *CycleGAN*. In the remaining examples, the styles in *CycleGAN*’s output are inconsistently applied, being either overly pronounced or insufficiently represented. Consequently, our framework demonstrates superior overall performance compared to *CycleGAN*. Furthermore, from a human perceptual perspective, the images generated by our method more closely resemble real-world appearances, highlighting its advantages over *CycleGAN*.

### 5.4. Quantitative Experiment

In this section, we provide a detailed quantitative analysis. During the course of our research, we utilized several backbone networks on this dataset (refer to Table 2 for details). To transfer the style, we employed GAN-based methods. The dataset includes multiple style and content pairs.

In the final results, we analyzed user assessments of image authenticity and, across multiple baselines, we obtained the highest approval ratings, exceeding 45% in both the single-image group and the 100-image group. Minor variations between the results of the two groups were observed, possibly because users considered certain features of the images (such as artifacts and color blocks), although the differences remain minimal. Our technology achieved higher confidence scores, outperforming the baseline *CycleGAN*, achieving a 133% improvement in single-pixel recognition and 195% improvement in recognizing the 100 generated images. We also analyzed the FCN scores for each image, dataset category, and class IoU. Ultimately, we concluded that our proposed method is effective. The single-pixel confidence, multi-class confidence, and IoU class categories are our three highest-scoring categories on the FCN. For single-pixel accuracy, our framework achieves approximately 129% of the baseline performance. For per-class pixel accuracy, we achieved approximately 241%, while the IoU per class reached around 236%. The comparison of these methods demonstrates that our strategy is highly effective for style transfer in unpaired photos.

**Figure 5:**
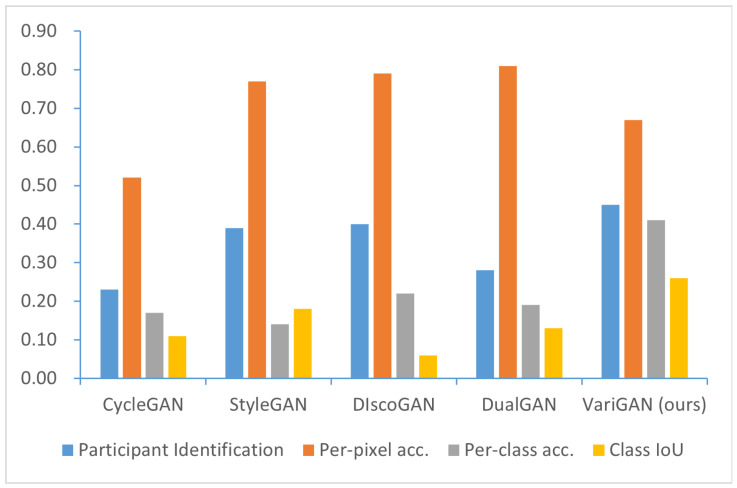
Comparison of the GAN-based models. The metrics we used included participant identification and FCN scores (per-pixel accuracy, per-class accuracy, and class IoU). *VariGAN* achieves the highest performance in participant identification. In FCN scores, *StyleGAN*, *DiscoGAN*, and *DualGAN* are specialized methods for face style transfer and achieve higher per-pixel accuracy scores compared to *VariGAN*. However, in other metrics, *VariGAN* performs comparably to or better than other GAN-based methods.

We also compared our framework, *VariGAN*, with several GAN-based approaches. The corresponding results are presented in Figure 5. *VariGAN* demonstrates superior performance in participant recognition tasks when compared to other methods. However, in terms of individual pixel accuracy, our framework achieves a relatively low score, as these GAN-based methods primarily emphasize facial style transfer. Most of the generated images consist of faces instead of diverse image categories. However, our method excels in the comparison of the other two indicators.

Although the above results show that the *VariGAN* framework is robust in terms of style transfer quality, the subsequent experiments indicate that *VariGAN* is computationally more expensive to train compared to other GAN-based methods. For a detailed analysis of this matter, see Section 5.7 and Section 7.

### 5.5. Ablation Experiment

The final step involves the ablation of each individual component. To evaluate whether UNet networks enhance generation quality, we adopted UNet networks as generators. To assess whether optimizing the loss function enhances the comprehensibility of generated images, we utilized LPIPS losses alone. To determine the role of the optimized discriminator, we employed the depthwise discriminator independently. For a quantitative analysis, see Table 3.

In terms of evaluation, using each component individually demonstrates better performance compared to *CycleGAN* alone (except when using the depthwise discriminator). Compared to *CycleGAN*, when using the UNet module alone accuracy improved by approximately 119% for individual images, 124% for each class classification, and 136% for class IoU. The optimized loss function resulted in approximately a 112% improvement in individual image accuracy, 135% improvement in image class accuracy, and 155% improvement in class IoU. The reason why the depthwise discriminator alone did not achieve significant improvement is likely due to the generating network’s limited capacity to constrain the generation process.

In addition, we performed a one-tailed test for the use of individual modules. The detailed analysis results are also displayed in Table 3. As shown in the table, except for the optimization of the depthwise discriminator, the other modules demonstrate significant improvements.

Although the depthwise discriminator network did not significantly enhance overall generation quality on its own, we observed a slight improvement in the quality of individual images, particularly in terms of accuracy per class. This module plays a crucial role in optimizing training by stabilizing the process and speeding up convergence. However, due to the limitations inherent in the generator in the current setup, the overall enhancement in generation quality remains limited. As shown by Section 5.7, the discriminator module significantly improves training efficiency by accelerating convergence. These findings underscore the module’s role as an auxiliary component in enhancing the model’s training dynamics, although its independent impact on the final output is limited.

### 5.6. Layer Choice of UNet Generator

**Figure 6:**
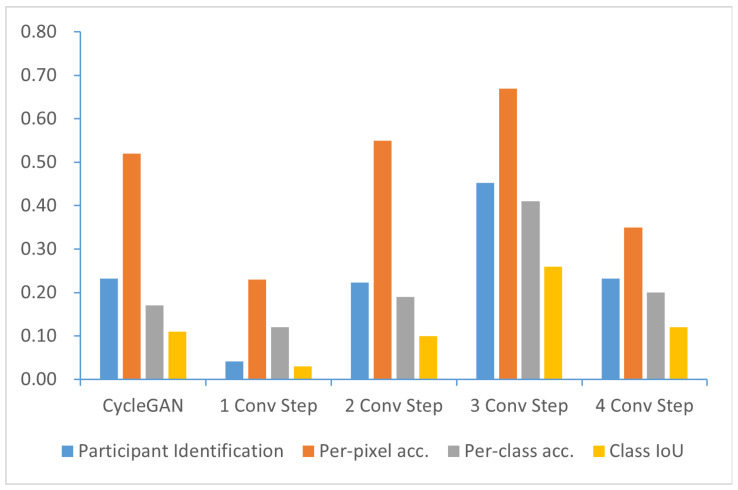
Comparison between different sampling steps based on participant identification ↑ and FCN scores ↑.

We conducted a quantitative experiment to evaluate the selection of sampling times for the generator, aiming to verify that the sampling times of the UNet generator chosen based on experience are appropriate. For the UNet generator used, 1,2,3, and 4 were chosen as the sampling times for each test, and the experiment was performed using the same dataset as the previous experiment. In the experiment, user identification and FCN score were still selected as evaluation indicators, and the results are shown in Figure 6.

The results show that style transfer is not as effective as *CycleGAN* when only one layer of sampling is used. When two-tier sampling is chosen, the FCN score reaches the level of *CycleGAN*, although user identification still lags behind *CycleGAN*. Using three layers of sampling yields the best results among all attempts. In this configuration, users achieve 45% confidence in identifying 100 images. For the FCN score, the pixel-wise accuracy reaches 0.67, the class-wise accuracy reaches 0.41, and the IoU class accuracy reaches 0.26. When four-layer sampling is used, the generation quality drops to around 23%, and the FCN score is essentially the same as *CycleGAN*. This confirms that selecting the three-layer UNet as the output of the generator network is the correct choice.

### 5.7. Qualitative Experiments on Training Efficiency of the Depthwise Discriminator

In Section 5.5, it was observed that the depthwise discriminator module does not enhance the generation quality of the framework. As can be seen from Table 4, following the improvements, the parameter count in the generator section and associated modules is significantly higher compared to *CycleGAN*. Generally, optimizing the discriminator can enhance training efficiency. To this end, the training durations of *CycleGAN* and our framework, both with and without the depthwise discriminator, were recorded. Since it is difficult to record the time for long training, only the training time for one epoch in each framework was recorded, and the total training time was calculated by multiplying the single-epoch time by the total number of epochs, and the results are shown in Table 5.

The results indicate that the *CycleGAN* framework has the shortest training time for the same dataset, while the longest training time is observed in *VariGAN* without the depthwise discriminator. When comparing *VariGAN* with and without the depthwise discriminator, it is evident that the depthwise module reduces training time and optimizes training efficiency. However, the training time remains lengthy due to the complexity of the generator module. This phenomenon is further demonstrated by the model parameters, as the generator parameters after optimization are over twice the size of the original generator parameters. To address this issue, future efforts should focus on developing more lightweight network architectures. However, in terms of inference time, our framework takes longer than *CycleGAN*. Nevertheless, the inference time for the relevant test set can still be ensured to be under half a minute.

### 5.8. An Analysis of the VariGAN Error Cases

Understanding *VariGAN*’s limitations is essential for identifying potential areas for improvement. This section analyzes model error cases in detail, focusing on image edges, generation style inconsistencies, and complex scenes. By examining these failure scenarios, we aim to identify the underlying causes and propose strategies for their resolution.

**Figure 7:**
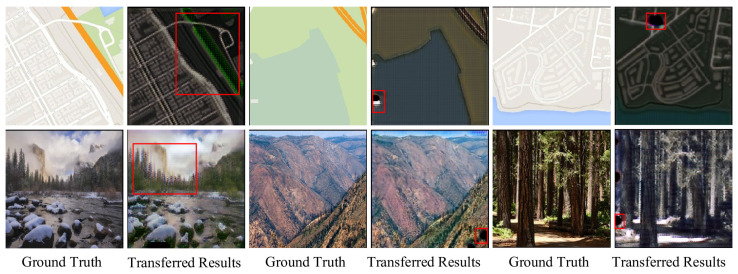
Failure cases in *VariGAN* generation are shown: Each set of images consists of the original image on the left and the corresponding generated image on the right. The failed regions are highlighted in red boxes. These failures are categorized into three types: edge artifacts, style transfer failures, and complex scene understanding failures.

Figure 7 presents examples of generation failures observed in *VariGAN*. These examples encompass the primary types of generation failures, including edge artifacts, style transfer failures, and interference in complex scenes. The examples focus on complex styles, such as maps and winter-to-summer transformations, to evaluate the framework’s performance under challenging conditions.

Edge artifacts may be attributed to the generator’s limited capacity to extract local features or the insufficient representation of edge regions in the dataset. To address this issue, feature extraction can be enhanced by incorporating edge consistency loss or employing a transformer-based generator framework. The failure of style conversion may be attributed to the limited size of the training dataset; this issue can be mitigated by expanding the dataset with additional images. For complex scene transformations, it is necessary to introduce a multi-scale generator or embed semantic feature hints into the images to enhance the generator’s perceptual capabilities.

## 6. Conclusions

In this study, we introduce *VariGAN*, a network specifically designed to improve the performance of GAN-based unpaired image style transfer. To enhance feature transfer, we utilize self-focused blocks and globally connected residual networks on top of a network of UNet generators to enhance the network’s capability. At the same time, in order to reduce inter-network dependency and improve training efficiency, we use a depth discriminator. This process proves more efficient when transferring styles from one image to another. We have conducted comprehensive experiments on a variety of datasets, and the results show that our method can effectively solve the problems of edge artifacts and style transfer failure in *CycleGAN*. Despite the longer time required for the training process, the inference speed remains acceptable in practical applications. For this purpose, our *VariGAN* can address the identified challenges.

## 7. Limitation and Future Work

Although our approach produced compelling results in numerous instances, the results were not consistently favorable. In certain instances involving complex colors or textures, the resulting images sometimes displayed edge artifacts or unwanted blotches. Style conversions sometimes failed, likely due to the complexity of the generator structure, which may lead to functional inconsistencies. Addressing these challenges by utilizing enhanced generators, such as Transformer-based feature extraction networks, and developing more robust attention mechanisms are priorities for future work. Furthermore, while our architecture produced higher-quality images compared to the baseline *CycleGAN*, this improvement required longer training times, exceeding one day for some datasets. Such extended training times were not observed in *CycleGAN*. To address this and enhance the practicality and deployment potential of our method, we can introduce knowledge distillation training logic to facilitate deployment on edge computing devices and reduce computing costs.

Our framework improves the quality of generation, but improving the framework’s generalization capabilities and developing a more lightweight network are top priorities. Additionally, scalability to high-resolution images continues to be a challenge. We plan to explore techniques such as progressive growing of GANs or multi-scale training approaches to handle larger image sizes efficiently. Furthermore, developing robustness against adversarial attacks is essential for real-world applications. Future work will focus on integrating adversarial training strategies or defensive distillation methods to bolster the framework’s resilience against such vulnerabilities. By addressing these issues, we aim to enhance the transparency and applicability of our approach in diverse settings.

## Figures and Tables

**Table 1:** The details of the datasets: Styles, the number of the images, and image sizes.

Dataset	TrainA	TrainB	TestA	TestB	Image Size
apple2orange	995	1019	266	248	256 × 256
horse2zebra	1067	1334	120	140	256 × 256
maps	1096	1096	1098	1098	600 × 600
winter2summer	1073	1073	210	210	256 × 256
vangogh2photo	400	6287	400	751	256 × 256
monet2photo	1072	6287	121	751	256 × 256
facades	400	400	106	106	256 × 256

**Table 2:** Comparison of different GAN variants in terms of participant identification and FCN (fully convolutional network) scores.

Participant Identification↑			FCN Score↑		
Loss	% Labeled Real (1 Image)	% Labeled Real (100 Images)	Per-Pixel Acc.	Per-Class Acc.	Class IoU
CoGAN [62]	4%	0.9%±0.5%	0.40	0.10	0.06
BiGAN/ALI [63]	8%	1.9%±0.9%	0.19	0.06	0.02
SimGAN [64]	8%	2.6%±1.1%	0.20	0.10	0.04
Feature loss + GAN	16%	0.3%±0.2%	0.06	0.04	0.01
DualGAN [17]	32%	33%±1.2%	0.27	0.13	0.06
cGAN [7]	20%	25%±4.6%	0.54	0.33	0.19
DRB-GAN [65]	12%	15%±0.2%	0.33	0.20	0.12
InST [66]	10%	11%±1.8%	0.49	0.20	0.03
CycleGAN [8]	36%	23.2%±3.4%	0.52	0.17	0.11
GAN Comp. [67]	52%	44%±2.5%	0.84	0.53	0.61
GauGAN [68]	54%	43%±3.8%	0.77	0.49	0.62
VariGAN (ours)	48%	45.2%±1.1%	**0.67**	**0.41**	**0.26**

**Table 3:** Ablation experiment: Comparison of different components in terms of FCN↑ (fully convolutional network) scores and BRISQUE↓. Significance measured using a one-tailed test. All are significant except depthwise discriminator method. * indicates that this value is an outlier.

Loss	Per-Pixel Acc.	Per-Class Acc.	Class IoU	One-Tailed Test	BRISQUE↓
CycleGAN	0.52	0.17	0.11	-	37.1
CycleGAN + UNet	0.62	0.21	0.15	3.65	27.8
CycleGAN + Depthwise	0.52	0.18	0.11	0.18 *	33.2
CycleGAN + LIPIS loss	0.58	0.23	0.17	3.56	30.8
VariGAN (ours)	0.67	0.41	0.26	7.78	24.2

**Table 4:** Statistics of total parameters of the models. All modules have input dimensions of 256×256.

	Generator	Discriminator	Self-Attention	ResNet
Total parameters (VariGAN)	39.23 M	0.84 M	0.10 M	2.95 M
Total parameters (CycleGAN)	11.38 M	2.76 M	–	1.18 M

**Table 5:** Comparison of *CycleGAN* and *VariGAN* with and without depthwise discriminator on different datasets based on training time (1 epoch time/total time), network parameters, and FLOPS. Overall number of epochs is 200.

Model	FLOPS	Parameters	apple2orange	horse2zebra	Maps	monet2photo	vangogh2photo
CycleGAN	58,541.74 M	14.14 M	167 s/556 min	218 s/729 min	179 s/598 min	997 s/3323 min	1003 s/3343 min
VariGAN-Depthwise	70,478.47 M	41.99 M	279 s/930 min	382 s/1273 min	294 s/980 min	1174 s/3913 min	1189 s/3963 min
VariGAN	69,009.22 M	40.07 M	257 s/857 min	336 s/1120 min	262 s/873 min	1053 s/3510 min	1066 s/3553 min

## Data Availability

The data that support the findings of this study are available from the corresponding author upon reasonable request. The source code is available at: https://github.com/Gdw040199/VariGAN.git (accessed on 4 March 2025).
